# Assessment of liver cancer biomarkers 

**Published:** 2020

**Authors:** Vahid Mansouri, Mohhamadreza Razzaghi, Abdolrahim Nikzamir, Alireza Ahmadzadeh, Majid Iranshahi, Mehrdad Haghazali, Mostafa Hamdieh

**Affiliations:** 1 *Proteomics Research Center, School of Rehabilitation, ShahidBeheshti University of Medical Sciences, Tehran, Iran*; 2 *Laser Application in Medical Sciences Research Center, ShahidBeheshti University of Medical Sciences, Tehran, Iran*; 3 *Faculty of Medicine, ShahidBeheshti University of Medical Sciences, Tehran, Iran*; 4 *Proteomics Research Center, Faculty of Paramedical Sciences, ShahidBeheshti University of Medical Sciences, Tehran, Iran*; 5 *Gastroenterology and Liver Diseases Research Center, Research Institute for Gastroenterology and Liver Diseases, Shahid Beheshti University of Medical Sciences, Tehran, Iran*; 6 *Rajaie Cardiovascular Medical and Research Center, Iran University of Medical Sciences, Tehran, Iran*; 7 *Department of Psychosomatic, Taleghani Hospital, Faculty of Medicine, ShahidBeheshti University of Medical Sciences, Tehran, Iran*

**Keywords:** Hepatocellular carcinoma, Biomarker, Diagnosis, Prognosis, Proteomics

## Abstract

Liver cancer is the third cause of cancer-related deaths in the world. It is primarily divides into two main types, namely hepatocellular carcinoma (HC) and cholangiocarcinoma (IC). Due to the increasing number of patients with liver cancer and the high mortality rate, early diagnosis of the disease can be helpful in treatment, but most patients are diagnosed atlate stages of HC. The aim of this study is to screen and provide an overview on candidate biomarkers related to primary liver cancer to introduce the critical ones. In this study, various biomarkers related to the diagnosis of primary liver cancer have been studied. Accordingly, biomarkers are divided into different groups as blood biomarkers classified as serum and plasma biomarkers, tissue biomarkers, microRNA biomarkers, proteomic biomarkers and altered genes. Previous researches have focused on liver cells and bile ducts, the surround cellular environment, how cells differentiate, and the types of genes expressed in liver cancer. Some even have focused on the origin of tumor cells and how they differentiate and develop. In all these studies, the expression of specific proteins and genes in liver cancer has been considered. Based on available sources, biomarkers can be considered as candidates to diagnose and prognosis of various types of primary liver cancer, from sources such as blood, tissue, mic-RNA, proteome and genes. However, more investigations are required to introduce a biomarker for precise detection of early liver cancer.

## Introduction

 Liver cancer with the fourth rank causes death in about 700,000 people annually (1). The most common type of liver cancer is called hepatocellular carcinoma (HC). There are several risk factors for hepatocellular carcinoma, some of which include hepatitis B and C viral infections, alcohol abuse, diabetes, autoimmune hepatitis, obesity and many metabolic diseases (2). Risk factors that lead to liver damage cause an inflammatory environmentand could induce the process of necrosis, tissue repair and chromosomal instability (3). Accumulation of inflammatory cytokines, reactive oxygen species and fibrosis leads to genetic and epigenetic changes resulting in hepatocellular carcinoma (3). Multikinaseinhibitors such as Sorafenib and Lenvatinib are currently approved drugs for advanced HC treatment but thesurvival rate of the patients treated with these drugs is not perfect yet,and new drug discovery is required for HC treatment (4, 5). HC initiation and progression is a multi-step process,butmolecular processes leading to HC formation is not completely understood (6). HC is a highly heterogeneous disease, and inter-tumor heterogeneity between different HCs has made effective treatment difficult (7). Target drug treatments are often given without considering the genetic history (8). HC has a stratified basis and unlike target therapies of other cancers with pre-stratified background, we need genetic background history for personalized medication(9). Genomic and trascriptomic studies have their own limitations, and changing them does not necessarily translated to protein levels lead to morphological differences (8). On the other hand, phosphorylation of proteins has not yet been studied for genomic profiling, which can regulate protein activity (8). Advancement in protein profiling technologies as proteomics are useful for identifying the molecular mechanisms of early hepatocellular carcinoma (10). This study investigates and extracts proteomics and genomics researches from valuable data banks to identify recent approaches to introduce new biomarkers related to different stages of HC.

**Primary Liver cancer**

Primary liver cancer generally include hepatocellular carcinoma(HC) with the incidence of 75-85%, intrahepatic cholangiocarcinoma(IC)with the incidence of 10-15%, and combination of both hepatocellular carcinoma and cholangiocarcinoma (HC-IC)(incidence of 1-4.4%)(11, 12). HC originates from hepatocytes as a malignant tumor and is the fifth common cancer cause(13). The second most common primary liver tumor is IC which originates from biliary epithelium(14) and the rare type of primary hepatic carcinoma originates from both hepatocytes and bile duct cells (15). There are different, sometimes confusing, biological behaviors for these cancers, and laboratory biomarker tests are safe and accurate to early diagnosis. Blood and histochemical biomarkers tracing could manage early monitoring and pathological classification of liver cancers to the prognosis and treatment of liver cancer patients. A-fetoprotein or AFT is the most commonbiomarker for primary liver cancer yet with low sensitivity (14). Therefore, the use and combination of different tumor biomarkers in order to diagnose primary liver cancer seems necessary to find new functional biomarkers according to prognosis judgments and treatment effect observations.

**Serum biomarkers**

Biomarkers in the blood are important for the early detection of primary liver cancer and they could be classified as proteins, cytokines, enzymes, and transcripts of dependent genes (16). According to the molecular characteristics of liver cancer biomarkers in the blood, they should not be used separately to diagnose different types of liver cancer and their combined use is necessary to diagnose these types of cancer (Table 1).

**AFP (a-fetoprotein)**

AFP is a glycoprotein biomarker derived from AFP is widely used in liver cancer diagnosis. However,it may increase in liver cirrhosis and hepatitis and AFP-L3 may increase in 20-30% of early HC(17). Sensitivity of AFP is 25% less than 3 cm in diameter for HC tumors and combined usage of AFP with other biomarkers can improve early diagnosis of HC(13). As AFP is significantly higher in IC patients compared to HC patients and does not change significantly in IC patients, it could be used as a powerful biomarker in IC diagnosis (15). Significant elevation of AFP in HC-IC patients is reported (18).

AFP-L3 is a fucosylated variant of AFP and derived only from tissues of tumors as a specific biomarker of HC. AFP-L3 specificity for early detection of HC was 90-95% (19). AFP-L3 demonstrates more sensitivity than AFP in early stages of HC,which could also reflectfeatures of tumors such as malignant invasion and little differentiation(20). Combination of AFP and AFP-L3 sensitivity in diagnosis of HC were significant (21), and AFP-L3 could be used as a supplementary test in low-level incidence of AFP in HC patients (16, 22).

**Table 1:** Blood Biomarkers involved in liver cancer

Biomarker	blood	Cancer type
AFU	Y	HC
AFP/AFP-L3	Y	HC
DCP	Y	HC
DCP & AFT	Y	HC
AFP	Y	HC-IC-HCIC
CA19-9	Y	ICC
AFP & OPN	Y	Small HC with negative AFP
GP73	Y	Early HC

AFP:a-fetoprotein, DCP: des-γ-carboxyprothrombin, AFU: a-L foucosidase, GP73: Golgi protein 73,OPN:Osteopontin,CA18-9: carbohydrate antigen19-9, GPC3: Glypcan-3

**Des** ***γ*** **carboxyprothrombin (DCP)**

DCP is a new serological biomarker produced by HC cells. DCP is elevated in HC patientsfollowing defects in carboxylation of prothrombin precursor after translation.As reported in a study, DCP was more sensitive than AFP(23). DCP were used as an effective biomarker to distinguish intra-hepatic metastasis and prognosis of HC in east Asian countries(24). Larger tumor size, more tumor numbers and bile duct invasions could be indicated by elevated levels of DCP, resulting inpoorer survival of patients(25). DCP could indicate HC in countries with high incidence of hepatitis B virus infections as East Asia and Africa(26). DCP and AFP combination in HC diagnosis with 2-4 cm tumors heightened sensitivity(27).

G**olgi protein 73 (GP73)**

GP73 areexpressed in the epithelial cells of different human tissues as a trans membrane glycoprotein of type II Golgi resident(28). GP73 is highly expressed in HC patients and moderately expressed in viral infections and cirrhosis(29). Expression and sensitivity of GP73 in HC is remarkably higher than AFP(30). GP73 suggested superior than AFP in early detection of HC(16, 30) .

**αLfucosidase or AFU**

AFU is an enzyme with the ability of fuco-glycocongugates degradation(31). Serum level of AFU in HC patients was higher than other patients with benign hepatic disorders(32). AFU is introduced as an early detecting biomarker for HC(33).AFU can be combined with AFP because itis positively linked with tumor size for early detection of HC (19).

**Carbohydrate antigen 199** (**CA19-9) **

CA19-9 is a general biomarker for the diagnosis of different types of adenocarcinoma as IC(36). CA19-9 is suggested for lymph node metastasis in IC patients (34). CA19-9 levels could be served as an individual prognostic biomarker for IC patients (35).

**Osteopontin(OPN)**

OPN is a phosphorylated extracellular protein binding to integrin. OPN is expressed in normal cells and tumors (36). Its level in patients diagnosed with hepatitis C viral was associated with HC, and was significantly higher than OPN level in chronic liver disease patients and healthy subjects (37).It seems that it is an early biomarker of HC.OPN elevated one year before HC diagnosis and in HC cases emerging from liver cirrhosis, OPN were indicated superior to AFP(37). It also induces macrophage activation.Tumor infiltration in OPN knockout HC mouse model through theactivation of colony stimulating factor 1 receptor pathway, leading to programmed deathwith ligand 1 expression in HC (38).

Concurrent detection of AFP,AFP-L3 and DCP in serum is used for early detection of HC at present (39). Elevated 3 biomarkers expression is accompanied with tumors invasiveness(39).AFP is a valuable biomarker for diagnosis and prognosis of HC, and the role of DCP and AFP-L3 in serum is supplement (16).

**Histological Biomarkers**

Histopathological methods for the diagnosis of liver cancer can be considered as a definitive method that is complemented by immunohistochemistry techniques. In this regard, biomarkers in cancer tissues can indicate the condition and morphology of tumor cells.Therefore, tissue biomarkers play an important role in the diagnosis and treatment of cancers and benign tumors of HC, IC, and HCIC.In the following, we introduce some major tissue biomarkers for the diagnosis of primary liver cancer.

**Heat shock protein 70 (HSP70)**

HSP70 expression is low in normal cases but in response to hypoxia, heat, genotoxic agents and food starvation, it is expressed significantly high(40).HSP70 gene is highly up-regulated in early HC tissue sections (41). Over expression of HSP70 isaccompanied with portal vein invasion (42). Another study revealed that HSP70 expression is associated with vascular invasion, cell proliferation, lymph node metastasis and larger tumor size in HC (43). However, over-expression of HSP70 could not demonstrate survival of patients with HC (42).

**Hepatocyteparaffin1(Hep-Par1)**

Hep-Par1 is ahighly sensitive monocolonal antibody for hepatocellular differentiation(44).It is associated withmitochondrial antigens of malignant and nonmalignant hepatic carcinoma cells (45). This biomarker is completely expressed in well differentiated HC but may be negative in poorly differentiated HC (46). Therefore, Hep-Par1 could be used for the diagnosis of poorly differentiated HC and liver cancer metastasis in combination with other biomarkers.

**Table 2:** Histopathological biomarkers for liver cancer

Biomarker	Tissue	Cancer Type
HSP70	Y	EarlyHC
GS	Y	HC
GPC3	Y	HC
Hep Par1	Y	HC
ARG1	Y	HC
CK19	Y	IC
CK7	Y	IC

HSP70:Heat shock protein70. GS: Glutaminsynthetase**GPC3: **glypican3.HepPar1: hepatocyte paraffin 1ARG1: arginase1. CK19:Cytokeratin19.CK7:Cytokeratin7 Y=Present-N=Absent

**Glypican3 (GPC3)**

GPC-3 is a heparan sulfateproteoglycan, and a member of glypican family (47). Itcan be lysed and released into serum as a soluble form of GPC-3 (48). GP can be considered as a specific biomarker of liver cancer, because its expression in these patients increases up to 80% and it can be used to diagnose HC from IC and other malignant tumors(49). Cell line investigations revealed that GPC-3 can modulate cell cycle progression, promote cell invasion, and migrationinHC cell cultures. It is also regarded asHC proprietary biomarker with high sensitivity of 97% (50). GPC-3 was introduced as a sensitive biomarker candidate for early detection of HC(51). As a strong histological biomarker for HC, it can also be considered as a serological biomarker. Research has shown that the rate of GPC-3 in the early occurrence of HC in serum is higher than AFP and can be used to early detection of HC (33).

**Arginase1 (Arg1)**

 Arg-1 is a sensitive biomarker for malignant & benign tumor cells of liver cancer and acts as an enzyme for arginine to ornithine hydrolysis (52). It is a suitable biomarker for hepatocellular differentiation too (53). Compared to Hep Par1, this enzyme is more sensitive in early HC differentiation, although Hep Par1 expression in non-hepatic tumors is also reported(54). Microarray analysis revealed that Arg-1 can be considered as a specific biomarker of HC (55). Therefore, itmay introduced as a specific candidate biomarker for HC (16).

**Cytokeratin or CK7**

CK7 is a member of cytokeratin family as intermediate filament. Itis expressed in epithelial cells and is mainly presented in gland ducts and absent in hepatocytes (56). The presence of CK7( similar to CK19) in gland ducts as bile ducts can justify its usage as a immunohistochemical biomarker to distinguish between IC and HC cells (57). Expression of CK7 and CK19 in IC patients is associated with aggressive tumor phenotypes and co-expression of CK7 & CK19 is considered as a top factor for independent prognosis of IC (58). The high- and low-level expressions of combined CK7 & CK19were respectively associated with low survival rate of patients (58). Therefore, CK7 & CK19 canbe assumed as a powerful biomarker for prognosis of IC but furtherresearch needs to confirm it.

**Glutamine synthetize** (**GS)**

GS catalyzes glutamate and ammonia to glutamine in the liver. It isexpressed in the hepatic veins beside hepatocytes in normal cases, but spreads on hepatocellular tumors (59). Glutamine provides energy for tumor cells and it is therefore diffused in HC cells and its expression is dependent on the WNT signal pathway. Itspositivity in liver cancer can also be associated with symptoms such as tumor size, cellular swelling, fatty liver and fibrosis (59). GC and HSP70 combined positive expression revealed well differentiated HC; therefore, two biomarkers expression may be a more efficienttool to distinguish between atypical neoplasms and well differentiated HC (60). Another study reported that immunoreactivity of GS and HSP70 could not identify IC cells origin properly (52). On the other hand, another study revealed that the specificity and sensitivity of GS in cirrhotic livers was significantly higher than non-cirrhotic livers (65). However, HSP70 and GS could not be identified in ICC origin tumors immunologically (66). Apparently, GC is an efficient tool for differentiating HC tracing(16). 

Biomarkers of hepatocytes commonly use GS, Arg-1,Hep-Par1and GPC-3. Bile duct cells biomarkers are CK7 and CK19. Biomarkers requiredistinguishing HC from ICC and separate different classes of HC as primary to metastatic.

**HCC involved genes**

Whole genome sequencing analysis of anHC related to hepatitis C viral infection were reported in 2011 for the first time to enter next-generation sequencing era (61). Sequencing researcheson HCC samples with different backgrounds have offered us novel insights. They have introducedviral host genomic interactions, gene mutations,epigenetic modifications and transcriptomic changes of HC (62-64). Frequently mutated genes as CTNNB1(B-catenin),TERT promoter and Tp53 havebeen explored recently as HC genomic alterations (65). Wheeler et al. suggested multiple genomic platforms and they believed in the central role of Sonic hedgehog signaling in HC (66). HC cells utilize dysregulation of phosphorylationmechanism to acquire cancer properties, butclassical genomic and proteomic analysis could not reflect these changes (8). HCC patients with Tuberous Sclerosis Complex (TSC) mutationhave a significant increase in S6 protein phosphorylation due to the activation ofmTOR kinase (67). Jiang et al. published a large-scale phosphoproteomics profiling study on early stages of HCC associated with hepatitis B virus. They stratified cohorts into three subtypes SI, SII and SIII tumors. Results demonstrated that TGF-B, HIF-1, Integrin and Rho GTPase pathways were up-regulated in SIII tumors with poor outcome after surgery and increased both AFP protein level and sterol O-acyletransfrase1 (SOAT1) expression (10). They concluded that SOAT1 is critical for maintaining cholesterol level to localize the trans-membrane receptors of HCC cells to growth and metastasis.Other researches in cholesterol maintenance revealed that loss of tumor suppressor factor p53 activates master transcription regulator of cholesterol synthesis pathway, and promotes maturation of sterol regulatory element binding protein 2 (SREBP2) for HCC development (68). However, liver cells with the lack of fatty acid synthase (FASN) utilize the cholesterol synthesis pathway,supporting c-MET oncogene mediated liver tumor formation through the regulation of SREBP2 (69). It seems that Cholesterol biosynthesis pathway is required for HCC development.In a proteomics study on hepatitis B leading to hepatocellular carcinoma, Gao et al.introduced the biomarkers of PYCR2 and ADH1A for metabolic programming in proteomic subgroups.They revealed CTNNB1 and TP53 mutation associated signaling and metabolic profiles withHC (70). PYCR2 is up-regulated in various types of cancer (71). ADH1A is involved in various xenobiotic substrates(72).Bioinformatics analysis of hepatocellular carcinoma biomarkers in patients revealed that FOXM1 was the most strongly connected gene amongup-regulated genes in PPI network (73). Other researchers suggested FOMX1 elevationin many tumors such as intrahepatic cholangiocarcinoma, esophageal adenocarcinoma, gastric cancer and HC (74-77). KIF4A gene expression couples with FOMX1 leads to excessive cell proliferation and promotes tumor development (77). Another study introduced FTCD as a core gene to distinguish early HC from benign tumors as a potential marker for early diagnosis of HC(78). Auto-immune hepatitis probability is 6 to 7 percent to induce HC (79). EPH2A mutations are frequent in IC and lymph node metastasis andangiogenesis was associated with the IC patients (80).

**MicroRNA biomarkers(miR)**

miRsare small RNA molecules usually containing 21-23 nucleotides which regulate gene expression through miR degradation or transitional repression(81).They areconsideredas a useful biomarkers for the diagnosis of HC and IC (82). Investigation of miRs in one study revealed that miR-25, miR-375 and Let7f could be significantly expressed in HC cells compared to the control group. Also, miR-122, miR-21, miR-192, miR223, miR27a, miR26a and miR-801 are introduced as specific biomarkers of liver cancer in comparison with healthy samples of cirrhosis and hepatitis B (83). Another study revealed that miR-122 and miR-21 are better for distinguishing HC (84). Between the two miRs, the miR122 had better performance because research in mice, lacking the miR122 gene, has prone to HC along with expression of AFP and IGF2 (85). In another study, miR-122 is significantly low in HC patients compared to Hepatitis C and control patients while the expression of miR-224 was significantly higher (86). It should be noted that the presence of miR-122 and miR-224 is associated with AFP, alkaline phosphatase level and tumor size,and they can be considered as biomarkers for early detection of liver cancer (86). Moshiri et al. used plasma RNA sequencing and found as HC biomarkersmiR-101, miR-1246 and miR-106b-3p in combination or individually (87). Hypoxia induced factors (HIF-1a and HIF-2a), miR-21 and miR-10b can be activated in acidic environment of HC to stimulate HC cells proliferation and migration (88).

**Proteomic study in HC**

Proteomic analysis as a powerful tool can uncover unidentified biomarkers in liver cancer with therapeutic potentials. Jiang et al. performed a large-scale proteomic profiling of early hepatocellular carcinoma associated with hepatitis B virus (8). They found that sterol O-acyltransferase 1 (SOAT1) expression is significantly high in liver cancer tumors the same as protein expression related to oncogenic pathways as integrin,Rho-GTBase, TFGF-B and hIF-1. Moon et al. revealed that loss of tumor suppressor p53 could promote maturation of sterol regulating binding protein 2 (SRBP-2) in liver cancer cells development (68). Another study mentioned that absence of fatty acid synthase in liver cells alternatively activate cholesterol synthesis pathways and up-regulation of SREBP2 (69). Therefore, it seems that cholesterol synthesis pathways lead to liver cancer. Nevertheless, more proteomic investigations will introduce suitable biomarkers for HC and IC according to metabolic pathways of cancer cells proliferation and migration.

**Signaling pathway biomarkers**

These biomarkers pathways contribute to the appearance and development of liver cancer. There are several signaling pathways associated with liver cancer formation and progression such as wnt, p53 signaling pathway, and c-Met. Canonical Wnt/β-catenin signaling pathway occurs in hepatocellular carcinoma through mutations in N terminal part of B-Catenin (89). Wnt signaling is active in most HCs (90). Combination of GPC-3 with Wnt leads to Wnt signaling pathway stimulation and localization of B-Catenin and liver carcinoma activation processes (48). Wnt presentation in tissues with hepatocellular carcinoma are reported in two classes named CTNNB1 and Wnt-TGF-B with different characteristics in liver carcinoma (91). Mutations in p53 signaling pathways may lead to HC. MDM2 is a transcriptional target and negative regulator of p53, and homeostasis between p53 and MDM2 as a feedback loop can control HC initiation and progression. With silencing p53 and over-expression of MDM2, hepatitis viruses can lost defense mechanisms of hepatocytes survival (92). c-Met signaling pathway is known with hepatocyte growth factor (HGF) and its receptor named mesenchymalepithelial transitional factor (c-Met). The pivot role of HGF-c-Met is in liver growth, regeneration and degeneration; however, c-Met inconsistent function may lead to the onset, proliferation and migration of HC*.*HGF-c-Met axis is a prognostic biomarker of HC(93). GPC-3 can control migration of HC cells via HGF-cMet pathway and heparin sulfate chain mediated growthfactor (94).

## Discussion

Biomarkers can be used for early detection ofliver cancer.In this respect, following the occurrence of various complications of the disease, and according to the expression of different biomarkers, appropriate diagnostic and therapeutic methods can be applied.On the other hand, it should be noted that many of the introduced biomarkers are not efficientand cancer patients should not be tested, because of subsequent complications (95). Nevertheless, biomarkers still play an important role in the diagnosis and prognosis of liver cancer. Early diagnosis of liver cancer depends on biomarker sensitivity and specificity.Serum biomarkers such as AFP are used to diagnose liver cancer in high-risk patientswith minimal invasiveness and rapid response.Combined use of biomarkers for early detection of liver cancer is prevalent. AFP, DPC and AFP-l3 biomarkers are usedin combination every six months for liver cancer detection (96). Recent studies introduced novel biomarkers for accurate diagnosis and early treatment of liver cancer such as AFU, GP73 and OPN. Biomarkers such asGP73, GPC3, AKR1B10 seem to be promising but require more validation (97). They have no more privilege over than AFP as demonstrates for osteopontin biomarker (98). Heterogeneity in meta-analysis approaches to HCC management with ultrasonography and AFP in patients of 14 countries with different and distinct outcomes justifies early cancer detection requirements (99). Mic-RNA can be evaluated as a diagnostic or prognostic tool or therapeutic target for liver cancer. However, inconsistency of assessed molecules measured in plasma and serum revealed discrepancy observed in researches, but mir-21 and mic-122 are promising as they were not differentially expressed in utilizing RNA sequencing (84). Moshiri et al. introduced some additional mic-RNAs, perhaps with more potential accuracy (87), but those observations remain preliminary and more investigation is required because of the lack of reproducibility of the findings.The use of immunohistochemical methods and H&E staining confirms the diagnosis of liver cancer, with routine histochemical biomarkers such as CPC-3, HSP-70, Hep Par1, CK7andArg-1 (16). Gene target therapies also indicated good curative influence. Genomics studies indicated positive CTNNB-1 & IDH potentials in HC & IC target therapies (100, 101) and gene target therapies can improve prognosis of liver cancer.

This study showed a variety of biomarkers related to different types of liver cancer. The new biomarkers will be put to clinical trials in the near future and open windows of hope for early detection and definitive treatment of liver cancer. Target drugs for some biomarkers may improve the survival rate of the liver cancer patients. Further studies on the signaling pathway in which biomarkers are involved will increase our knowledge of the molecular mechanism of the progression of liver cancer. Significant advances in technology are encouraging more researchers to use those advances to better identify cancer biomarkers to find a definitive and early treatment for the liver cancer.

Despite numerous studies and the introduction of numerous biomarkers, it seems that a specific biomarker that has the ability to detect liver cancer early along with AFP has not been introduced yet. Further clinical trials in different centers are required to specify the risk of liver cancer in various populations. Comprehensive data are needed to decide and select the appropriate biomarker, and future technological advances will help achieve this goal.

## Conflict of interests

The authors declare that they have no conflict of interest.
